# Supplementary material for: Proline Oxidation Supports Mitochondrial ATP Production When Complex I Is Inhibited
Source: Int J Mol Sci. 2022 May 4;23(9):5111. doi: 10.3390/ijms23095111 (PMC9106064; doi:10.3390/ijms23095111)
Supplement: Supplementary file 1 [file ijms-23-05111-s001.zip › Supplementary material (supplementary figures and their legends)_IJMS.pdf]

Proline Oxidation Supports Mitochondrial ATP Production When Complex I Is Inhibited

Gergely Pallag[1], Sara Nazarian[1], Dora Ravasz[1], David Bui[1], Timea Komlódi[2], Carolina Doerrier[2], Erich Gnaiger[2], Thomas N Seyfried[3], Christos Chinopoulos[1*]

[1] Department of Biochemistry and Molecular Biology, Semmelweis University, Budapest 1094, Hungary

[2] Oroboros Instruments, Innsbruck, Austria

[3] Biology Department, Boston College, Chestnut Hill, MA 02467, USA

* Corresponding author: chinopoulos.christos@eok.sote.hu

Supplementary Material

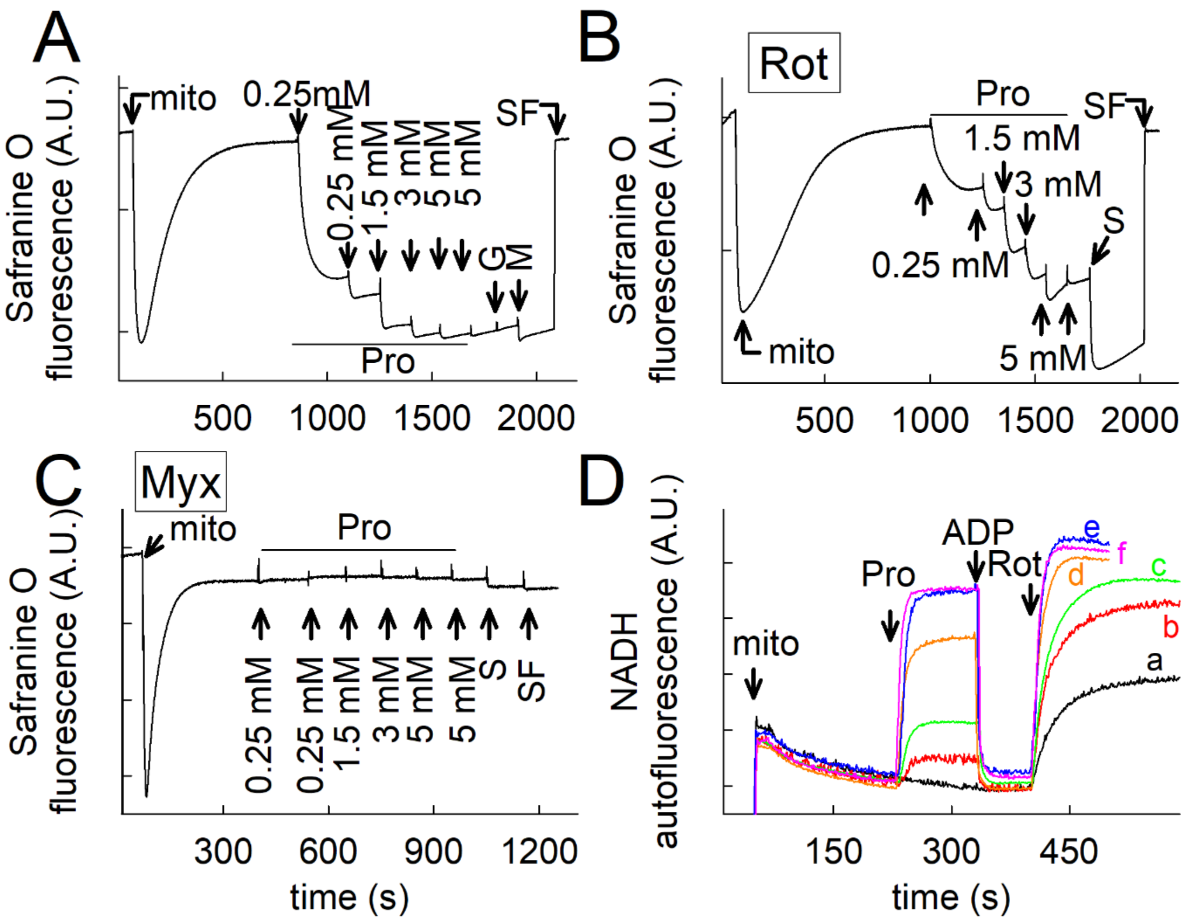

Supplementary Figure S1. Effect of proline concentration on $\Delta\Psi_{mt}$ and NADH autofluorescence of isolated mouse kidney mitochondria. No substrates were present prior to addition of mitochondria. **(A-C)** Time course of safranine O signal indicative of $\Delta\Psi_{mt}$ (arbitrary units A.U.). Glutamate (G), malate (M), succinate (S), 1 μM SF and proline (Pro) added at the concentrations where indicated. **(A)** No targeted inhibition of electron transfer pathways. **(B)** Rotenone prior to addition of mitochondria. **(C)** Myxothiazol (Myx) prior to addition of mitochondria. **(D)** Time course of NADH autofluorescence (arbitrary units A.U.). Concentrations of Pro: black (a): 0 mM, red (b): 0.25 mM, green (c): 0.5 mM, orange (d): 2 mM, blue (e): 5 mM, magenta (f): 10 mM.

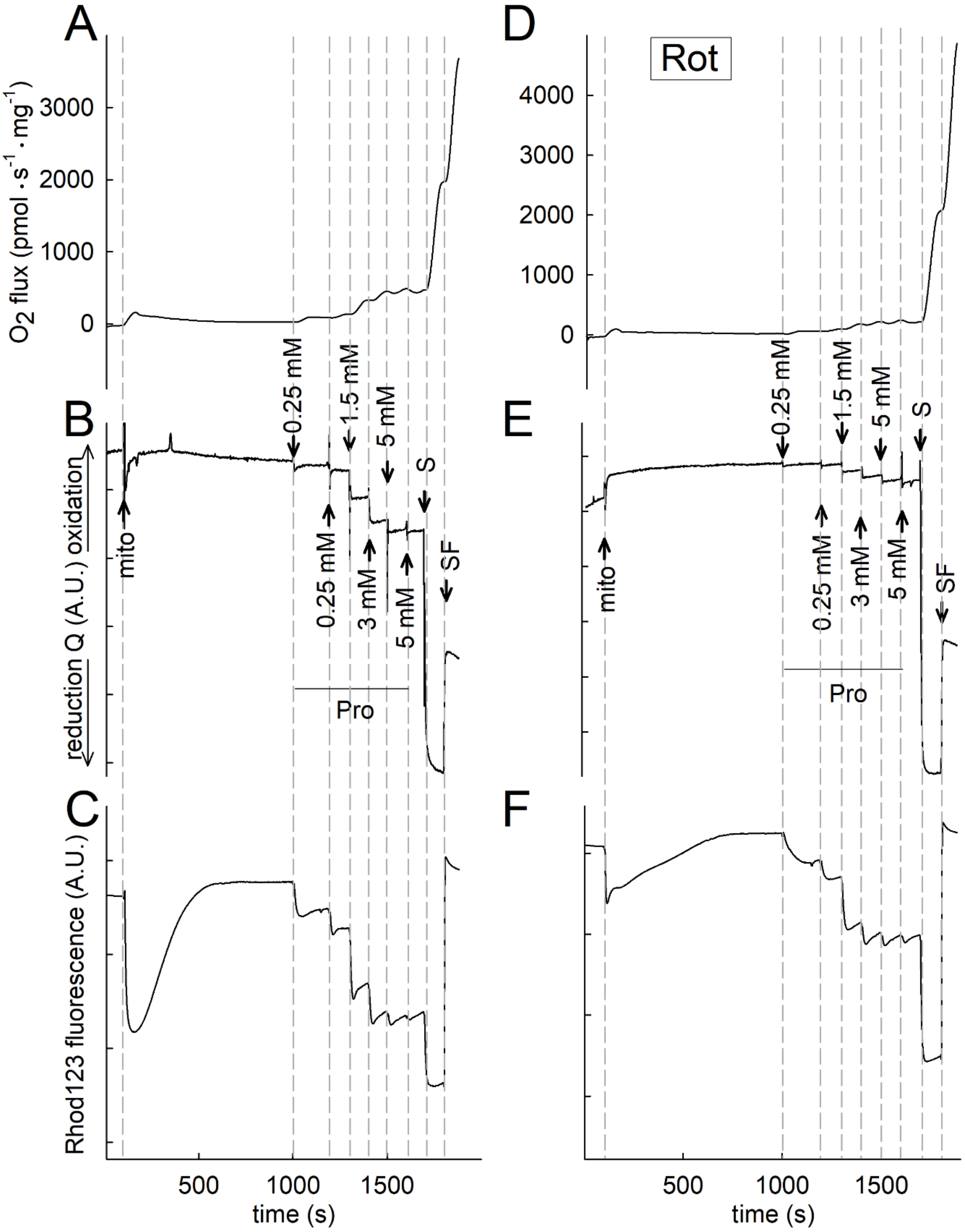

Supplementary Figure S2. Coenzyme Q (Q) redox state recorded simultaneously with oxygen consumption rate and rhodamine 123 fluorescence in isolated mouse kidney mitochondria using the NextGen-O2k. No substrates were present before addition of mitochondria; proline (Pro) added at the concentrations indicated; succinate (S, 5 mM); 0.25 µM SF. **(A-C)** Time course of oxygen consumption, Q redox state, and rhodamine 123 fluorescence indicative of $\Delta\Psi_{mt}$ (arbitrary units A.U.), respectively. **(D-F)** As in A, B, and C, but with rotenone in the medium.

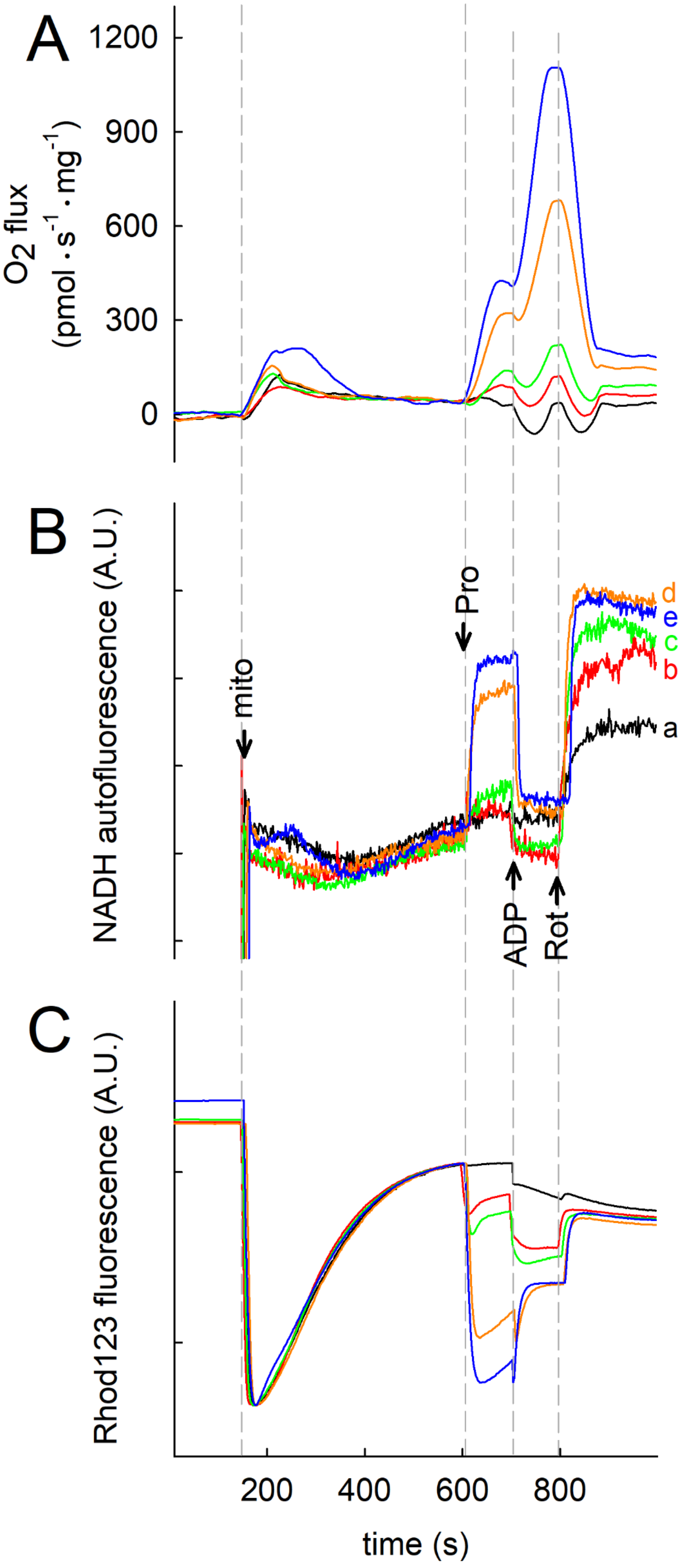

A
1200
900
600
300
0
O2 flux
(pmol·s-1·mg-1)
B
NADH autofluorescence (A.U.)
mito
Pro
ADP
Rot
d
e
c
b
a
C
Rhod123 fluorescence (A.U.)
200
400
600
800
time (s)

Supplementary Figure S3. Effect of proline concentration on respiration, $\Delta\Psi_{mt}$, and NADH autofluorescence of isolated mouse kidney mitochondria. Time courses of **(A)** oxygen consumption, **(B)** NADH autofluorescence, and **(C)** rhodamine 123 fluorescence indicative of $\Delta\Psi_{mt}$ (arbitrary units A.U.), recorded simultaneously from the same kidney mitochondria using the NextGen-O2k and aligned on the dashed grey lines. No substrates were present prior to addition of mitochondria. Concentrations of proline (Pro): black (a): 0 mM, red (b): 0.25 mM, green (c): 0.5 mM, orange (d): 2 mM, blue (e): 5 mM.

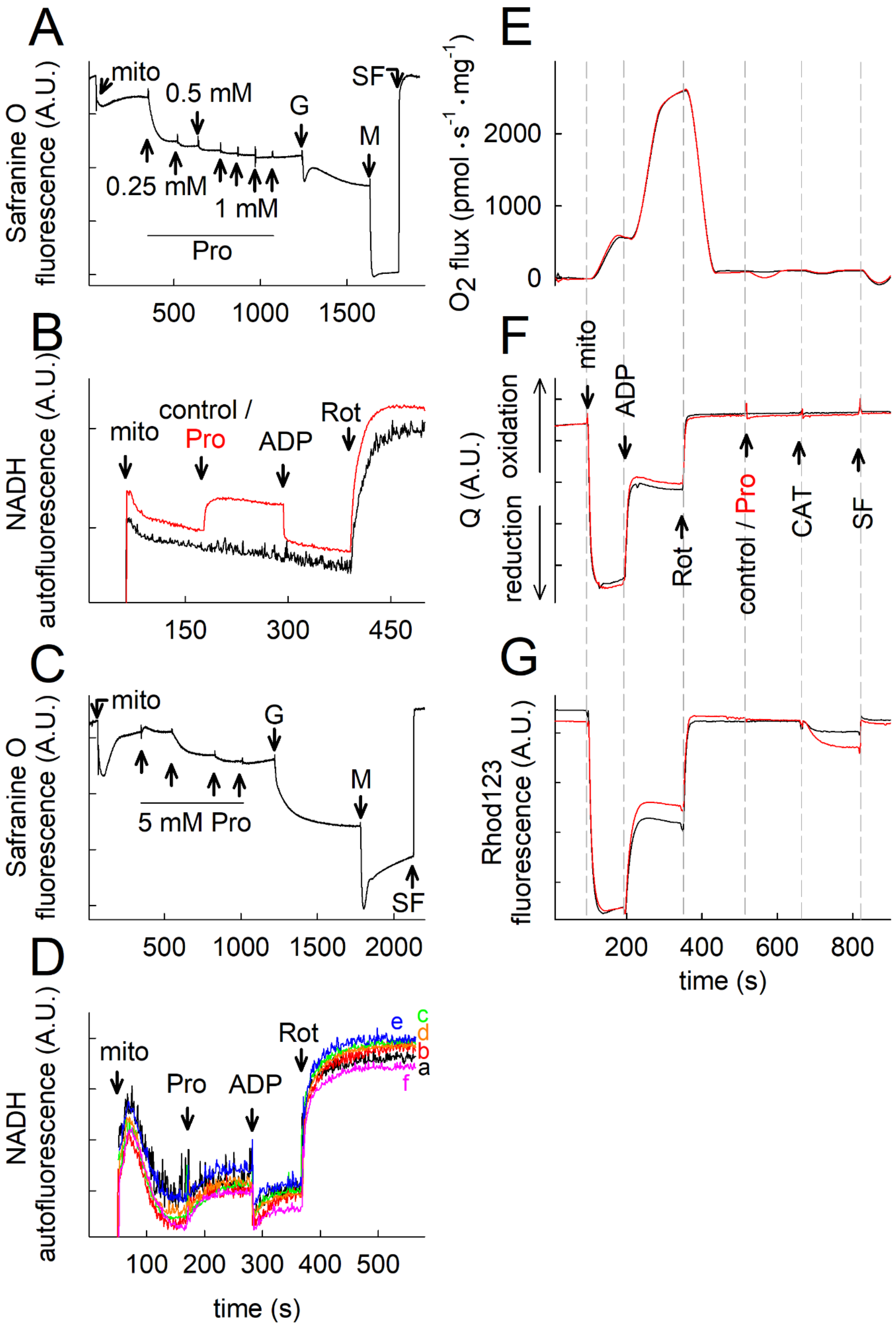

Supplementary Figure S4. Effect of proline concentration on $\Delta\Psi_{mt}$ and NADH autofluorescence of mitochondria isolated from mouse brain (A, B) and mouse heart

(C, D). No substrates were present prior to addition of mitochondria; proline (Pro) added at the concentrations where indicated. **(A)** Time course of safranine O signal indicative of $\Delta\Psi_{mt}$ (arbitrary units A.U.). Glutamate (G) and malate (M) added where indicated. 1 µM SF. **(B)** Time course of NADH autofluorescence (arbitrary units A.U.). Red trace: 10 mM proline. **(C)** Time course of safranine O signal indicative of $\Delta\Psi_{mt}$ (arbitrary units A.U.). Glutamate (G) and malate (M) added where indicated. 1 µM SF. **(D)** Time course of NADH autofluorescence (arbitrary units A.U.). Pro: black (a): 0 mM, red (b): 1 mM, green (c): 2 mM, orange (d): 5 mM, blue (e): 10 mM, magenta (f): 20 mM. **(E-G)** Time courses of oxygen consumption, Q redox state and rhodamine 123 fluorescence indicative of $\Delta\Psi_{mt}$ (arbitrary units A.U.), respectively, recorded simultaneously in the same brain mitochondria using the NextGen-O2k and aligned on the dashed grey lines. 0.25 µM SF. Black trace: GM; red trace: GM and 5 mM Pro.

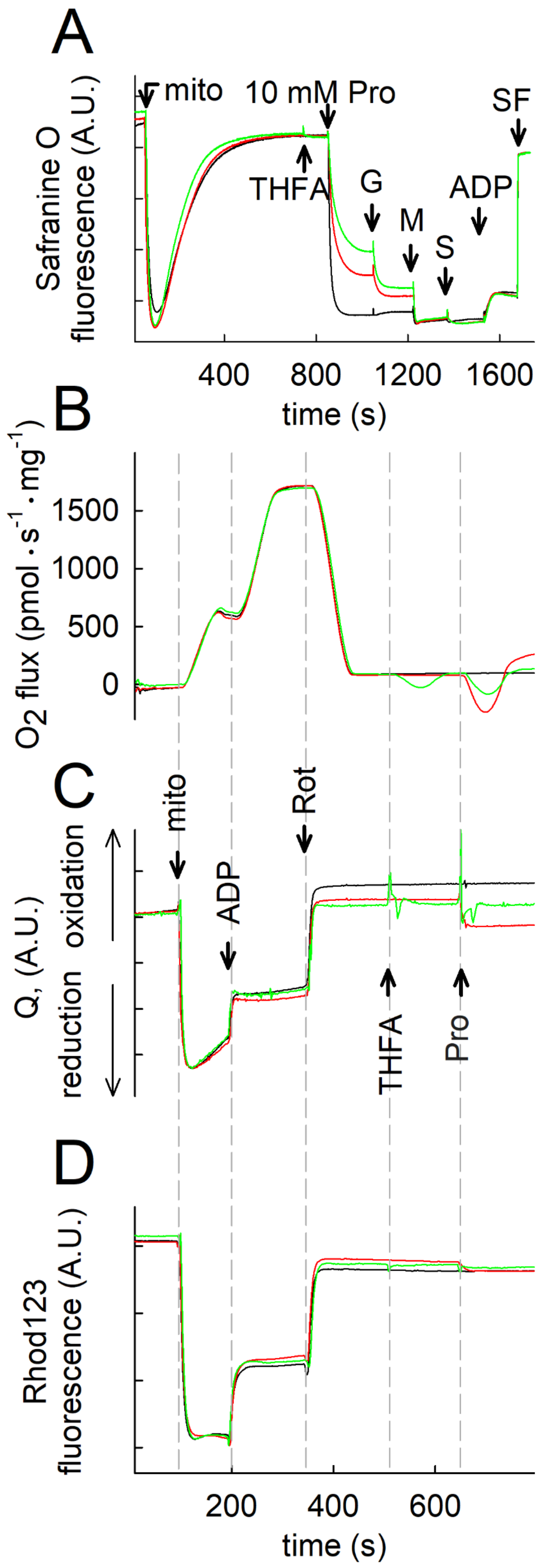

A
mito
10 mM Pro
THFA
G
M
S
ADP
SF
Safranine O fluorescence (A.U.)
400
800
1200
1600
time (s)
B
O2 flux (pmol · s-1 · mg-1)
1500
1000
500
0
C
mito
ADP
Rot
THFA
Pro
Q, (A.U.)
reduction oxidation
D
Rhod123 fluorescence (A.U.)
200
400
600
time (s)

Supplementary Figure S5. The effects of THFA on kidney mitochondria. (A) Time course of safranine O signal indicative of $\Delta\Psi_{mt}$ (arbitrary units, A.U.) in isolated kidney mitochondria. No substrates were present in the buffer prior to addition of mitochondria. Proline (Pro, 10 mM), glutamate (G), malate (M), succinate (S), ADP and 1 µM SF added where indicated. Red trace: plus 5 mM THFA; green trace: plus 10 mM THFA. (**B-D**) Time courses of oxygen consumption, Q redox state and rhodamine 123 fluorescence indicative of $\Delta\Psi_{mt}$ (arbitrary units A.U.), respectively, recorded simultaneously from the same kidney mitochondria using the NextGen-O2k and aligned on the dashed grey lines. GM and itaconate (10 mM) always present. Black traces: GM and itaconate (10 mM), red traces: plus 10 mM Pro; green traces: plus 10 mM proline and 10 mM THFA where indicated.

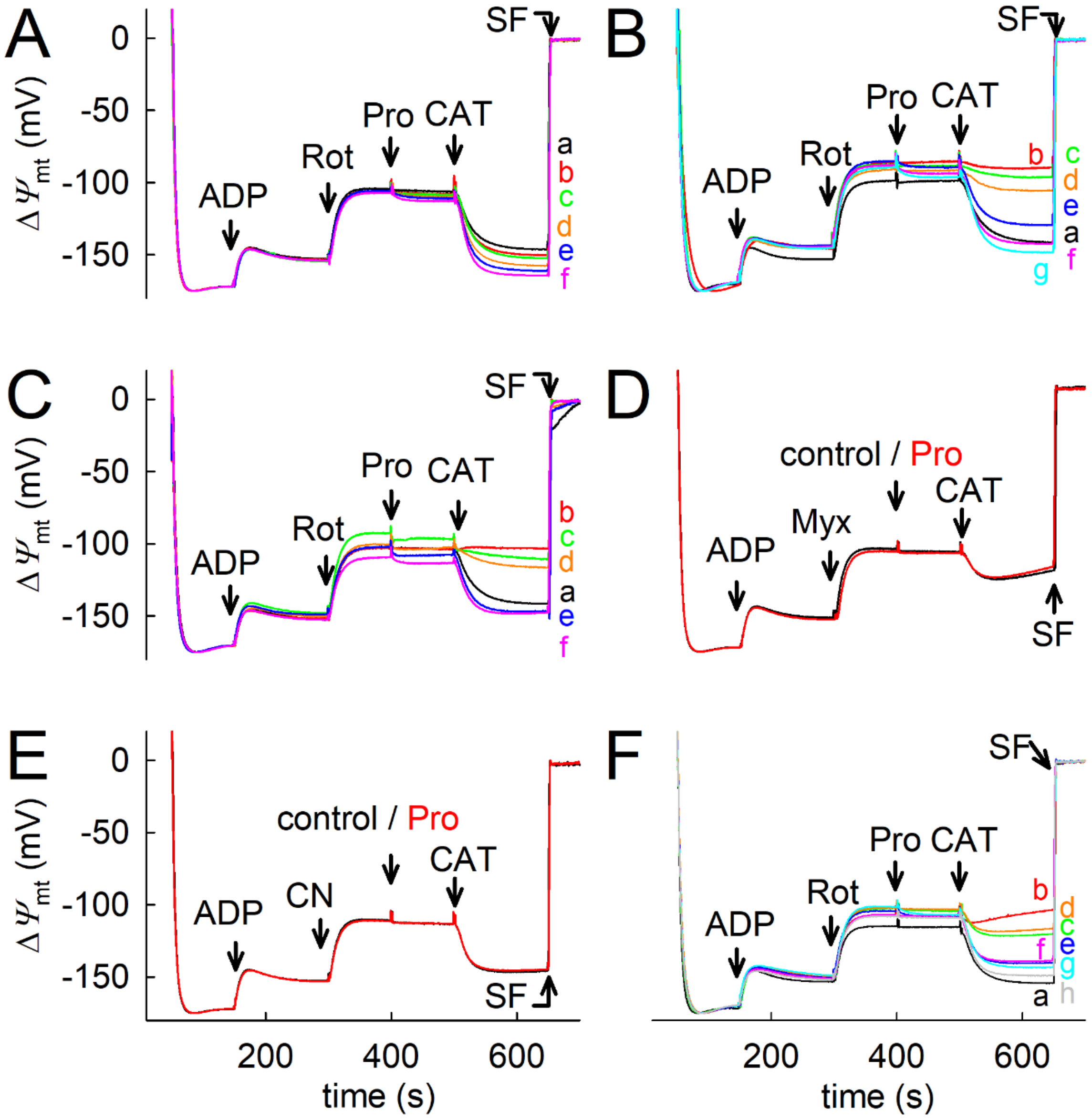

Supplementary Figure S6. The effect of proline on ANT directionality in isolated mouse kidney mitochondria as a function of targeted inhibition of electron transfer pathways using various substrate-inhibitor combinations. Traces are time courses of safranine O signal calibrated to $\Delta\Psi_{mt}$. GM always contained in the medium. Itaconate (Itac, 5 mM), ketobutyrate (2 mM) where indicated. **(A)** Black (a): GM; red (b): GM and 0.25 mM proline

(Pro); green (c): GM and 0.5 mM Pro; orange (d): GM and 2 mM Pro; blue (e): GM and 5 mM Pro; magenta (f): GM and 10 mM Pro. **(B)** Black (a): GM; red (b): GM & itaconate; green (c): GM & itaconate and 0.25 mM Pro; orange (d): GM & itaconate and 0.5 mM Pro; blue (e): GM & itaconate and 2 mM Pro; magenta (f): GM & itaconate and 5 mM Pro; cyan (g): GM & itaconate and 10 mM Pro. **(C)** Black (a): GM; red (b): GM & ketobutyrate; green (c): GM & ketobutyrate and 0.25 mM Pro; orange (d): GM & ketobutyrate and 0.5 mM Pro; blue (e): GM & ketobutyrate and 2 mM Pro; magenta (f): GM & ketobutyrate and 5 mM proline. **(D)** Black trace: GM; red trace: GM and 10 mM proline added after myxothiazol (Myx). **(E)** Black trace: GM; red trace: GM and 10 mM proline added after NaCN (CN, 1 mM). **(F)** As in B, but in traces d, f and h proline was present in the media prior to addition of mitochondria. Black (a): GM present in the buffer; red (b): GM and Itac present in the buffer; green (c): GM and Itac present in the buffer, 0.5 mM Pro added at 400 s; orange (d): GM, Itac and 0.5 mM Pro present in the buffer from the begining; blue (e): GM and Itac present in the buffer; 2mM Pro added at 400 s; magenta (f): GM, Itac and 2 mM Pro present in the buffer from the beginning; cyan (g): GM and Itac present in the buffer, 5 mM Pro added at 400 s; silver (h): GM, Itac and 5 mM Pro present in the buffer from the beginning.

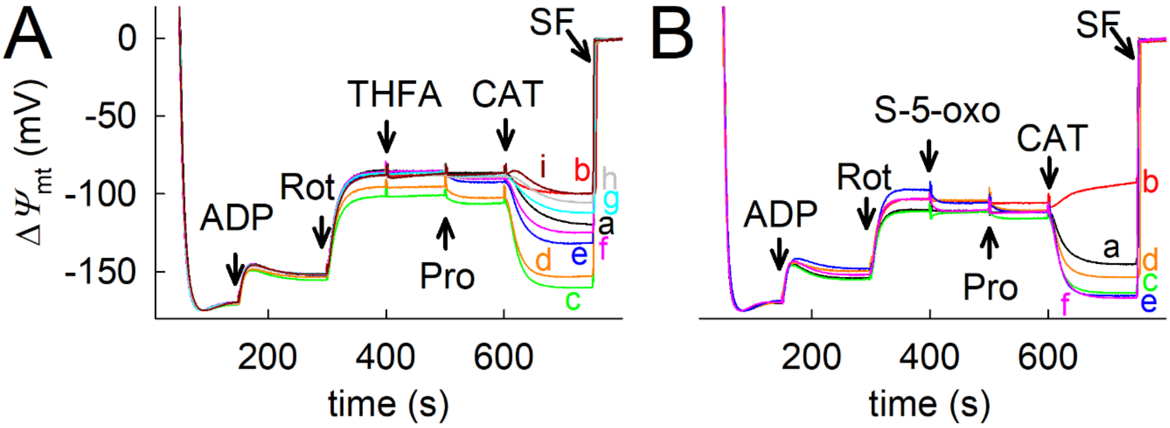

Supplementary Figure S7. The effect of THFA and S-5-oxo on proline-induced changes of ANT directionality in isolated mouse kidney mitochondria. Traces are time courses of safranine O signal calibrated to $\Delta\Psi_{mt}$. GM always contained in the medium. Proline (Pro, 10 mM) where indicated. **(A)** Itaconate (2 mM) where indicated. Black (a): GM; red (b): GM & itaconate; green (c): GM & proline; orange (d): GM & proline & itaconate; blue (e): GM & proline & itaconate and 1 mM THFA; magenta (f): GM & proline & itaconate and 2 mM THFA; cyan (g): GM & proline & itaconate and 5 mM THFA; grey (h): GM & proline & itaconate and 10 mM THFA; brown (i): GM & proline & itaconate and 15 mM THFA. **(B)** Itaconate (6 mM) where indicated. Black (a): GM; red (b): GM & itaconate; green (c): GM & proline; orange (d): GM & proline & itaconate; blue (e): GM & proline & itaconate and 10 mM S-5-oxo; magenta (f): GM and 10 mM S-5-oxo.

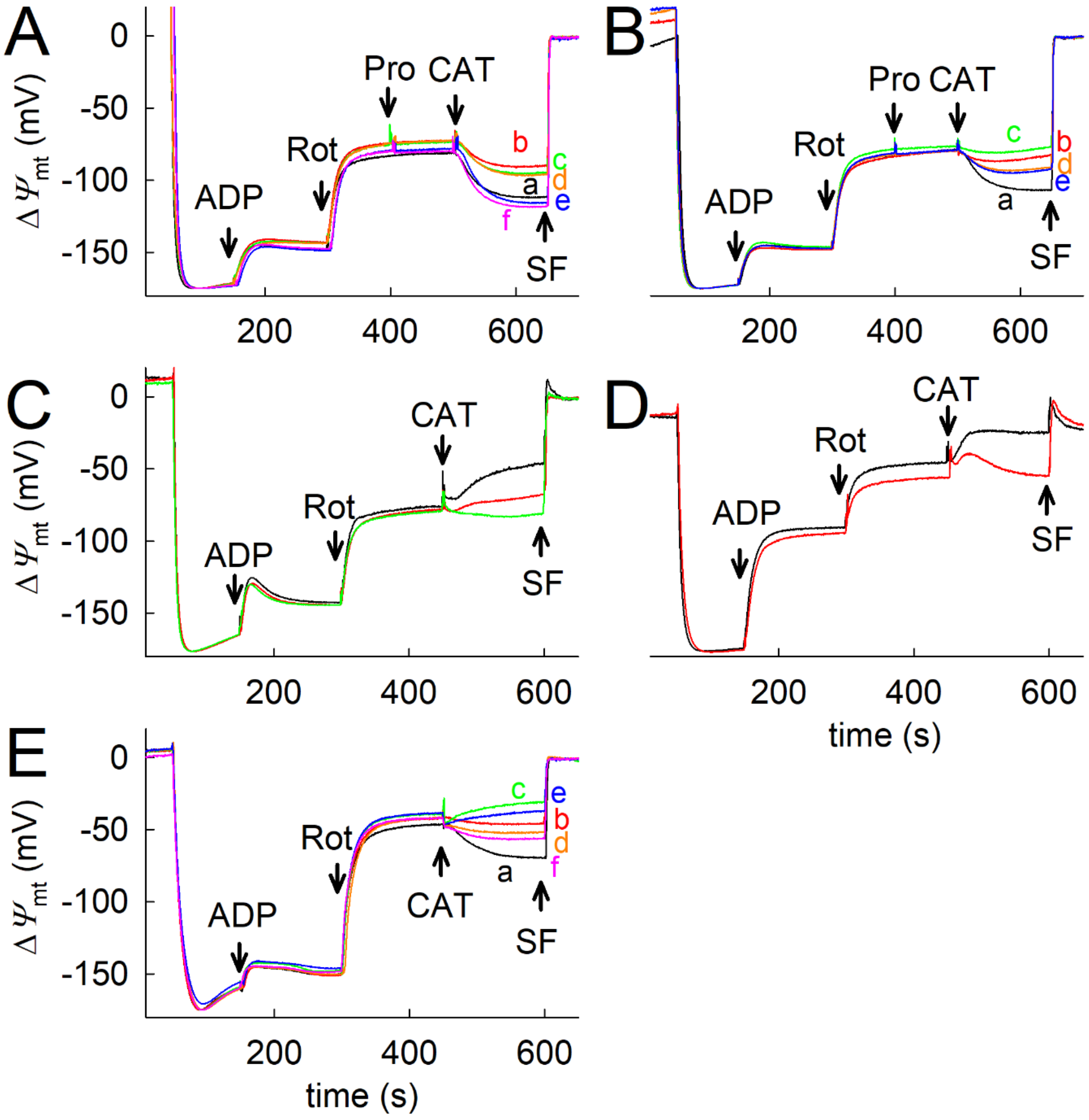

Supplementary Figure S8. The effect of proline on ANT directionality in isolated mouse brain mitochondria. Traces are time courses of safranine O signal calibrated to $\Delta\Psi_{mt}$. Pyruvate (P, 5 mM), glutamate (G), malate (M), βOH (2 mM), itaconate (2 mM) where indicated. **(A)** Black (a): GM; red (b): GM and 0.25 mM proline (Pro); green (c): GM and 0.5 mM Pro; orange (d): GM and 2 mM Pro; blue (e): GM and 5 mM Pro; magenta (f): GM and 10 mM Pro. **(B)** Black (a): GM; red (b): GM & itaconate; green (c): GM & itaconate and 2 mM Pro; orange (d): GM & itaconate (2 mM) and 5 mM Pro; blue (e): GM & itaconate and 10 mM Pro. **(C)** Black trace: G; red trace: G & 5 mM Pro; green trace: G & 10 mM Pro. **(D)** Black trace: P; red trace: P & 5 mM Pro. **(E)** Black (a): GM; red (b): GMβOH; green (c): GMβOH & 0.25 mM Pro; orange (d): GMβOH & 0.5 mM Pro; blue (e): GMβOH & 2 mM Pro; magenta (f): GMβOH & 5 mM Pro.

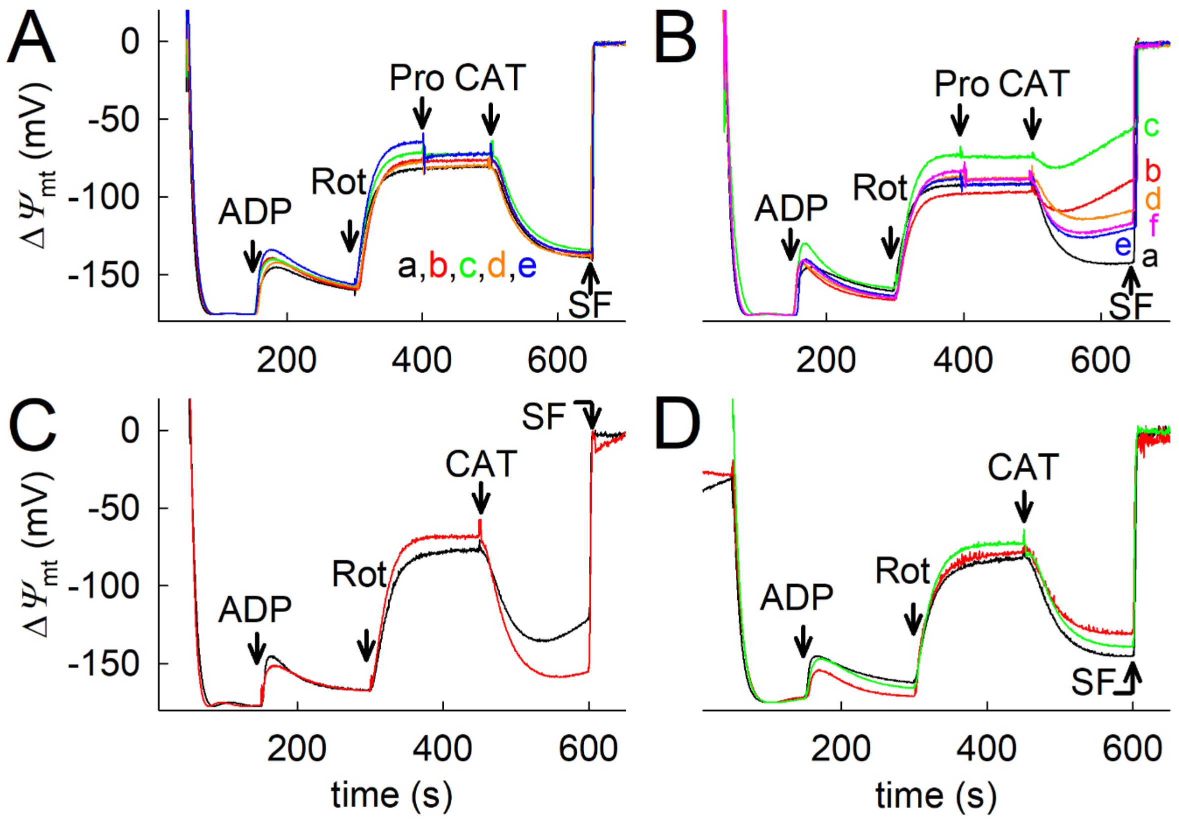

Supplementary Figure S9. The effect of proline on ANT directionality in isolated mouse heart mitochondria. Traces are time courses of safranine O signal calibrated to $\Delta\Psi_{mt}$. βOH (5 mM), where indicated. **(A)** GM always contained in the medium, increasing concentrations of proline (Pro); black (a): 0 mM; red (b): 5 mM; green (c): 10 mM; orange (d): 20 mM; blue (e): 40 mM. **(B)** GM always present in the medium, 5 mM itaconate (Itac) present in the medium when indicated, Pro added where indicated. Black trace (a): GM; red (b): GM and Itac; green (c): GM, Itac and 5 mM Pro; orange (d): GM, Itac and 10 mM Pro; blue (e): GM, Itac and 20 mM Pro; magenta (f): GM, Itac and 30 mM Pro. **(C)** Black trace: G; red trace: G & 5 mM Pro. **(D)** Black trace: GM; red trace: GMβOH; green trace: GMβOH & 5 mM Pro.

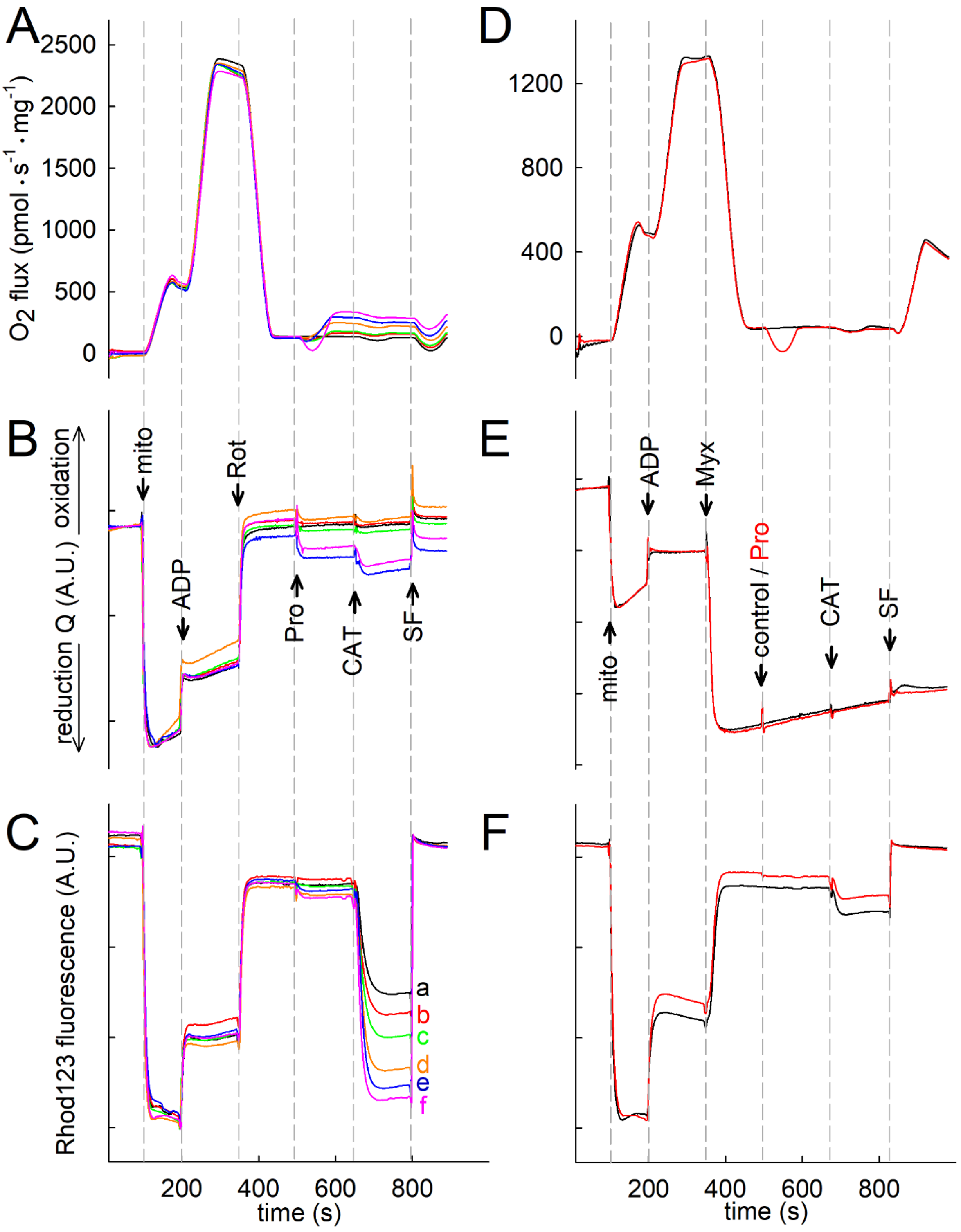

Supplementary Figure S10. The effect of proline on CAT-induced changes in Q redox state and its abolition by myxothiazol. Oxygen consumption rate **(A, D)**, Q redox state **(B, E)**, and rhodamine 123 fluorescence **(C, F)** indicative of $\Delta\Psi_{mt}$ (arbitrary units A.U.) measured simultaneously in the same mouse kidney mitochondria using the NextGen-O2k and aligned on the dashed grey lines. 0.25 µM SF. **(A-C)** Rotenone (Rot), GM and increasing concentrations of Pro; black (a): 0 mM; red (b): 0.25 mM; green (c): 0.5 mM; orange (d): 2 mM; blue (e): 5 mM; magenta (f): 10 mM. **(D-F)** Myxothiazol (Myx, 0.1 µM). Black traces: GM & 10 mM itaconate; red traces: 10 mM Pro added where indicated.

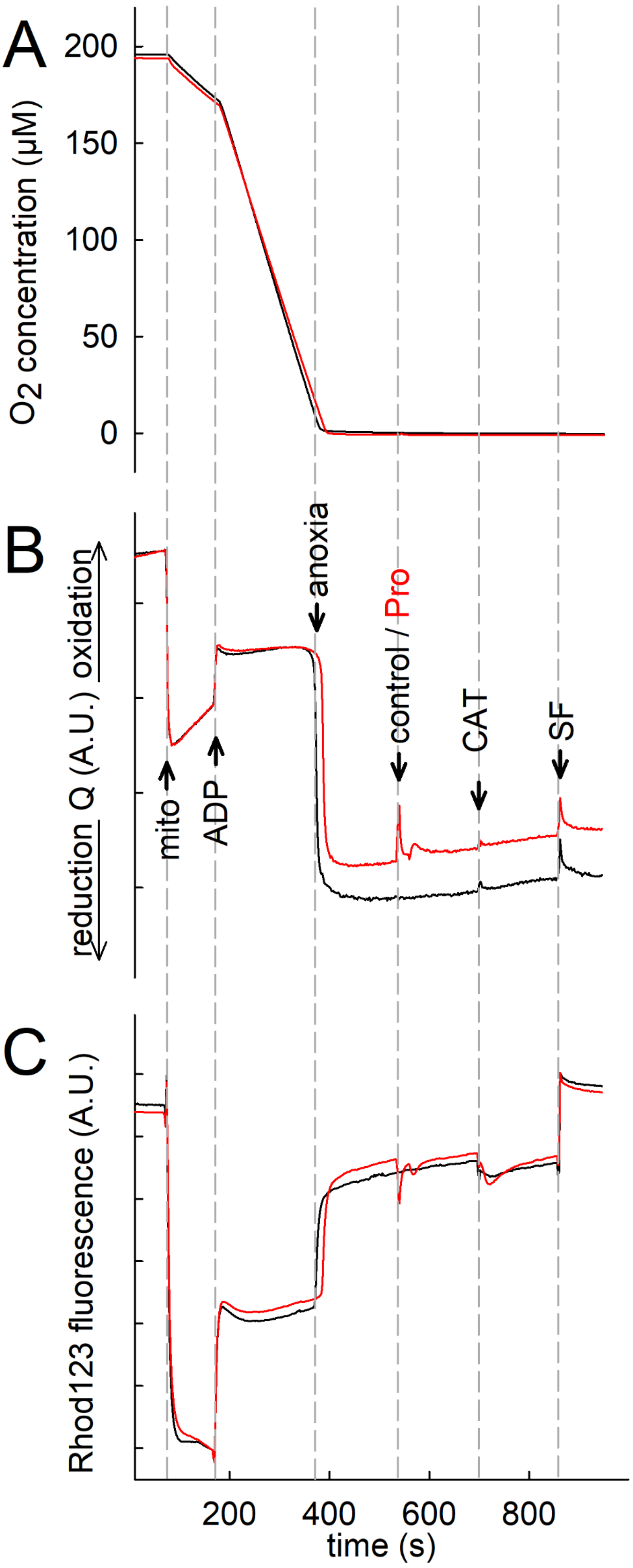
A
B
C
200
150
100
50
0
O2 concentration (μM)
reduction Q (A.U.) oxidation
Rhod123 fluorescence (A.U.)
mito
ADP
anoxia
control / Pro
CAT
SF
200
400
600
800
time (s)

Supplementary Figure S11. No effect of proline on Q redox state of anoxic kidney mitochondria. Oxygen concentration **(A)**, Q redox state **(B)**, and rhodamine 123 fluorescence **(C)** indicative of $\Delta\Psi_{mt}$ (arbitrary units A.U.) measured simultaneously in the same mouse kidney mitochondria using the NextGen-O2k and aligned on the dashed grey lines. Black traces: GM & 5 mM itaconate; red traces: GM & 5 mM itaconate plus 10 mM proline added where indicated. 0.25 µM SF.

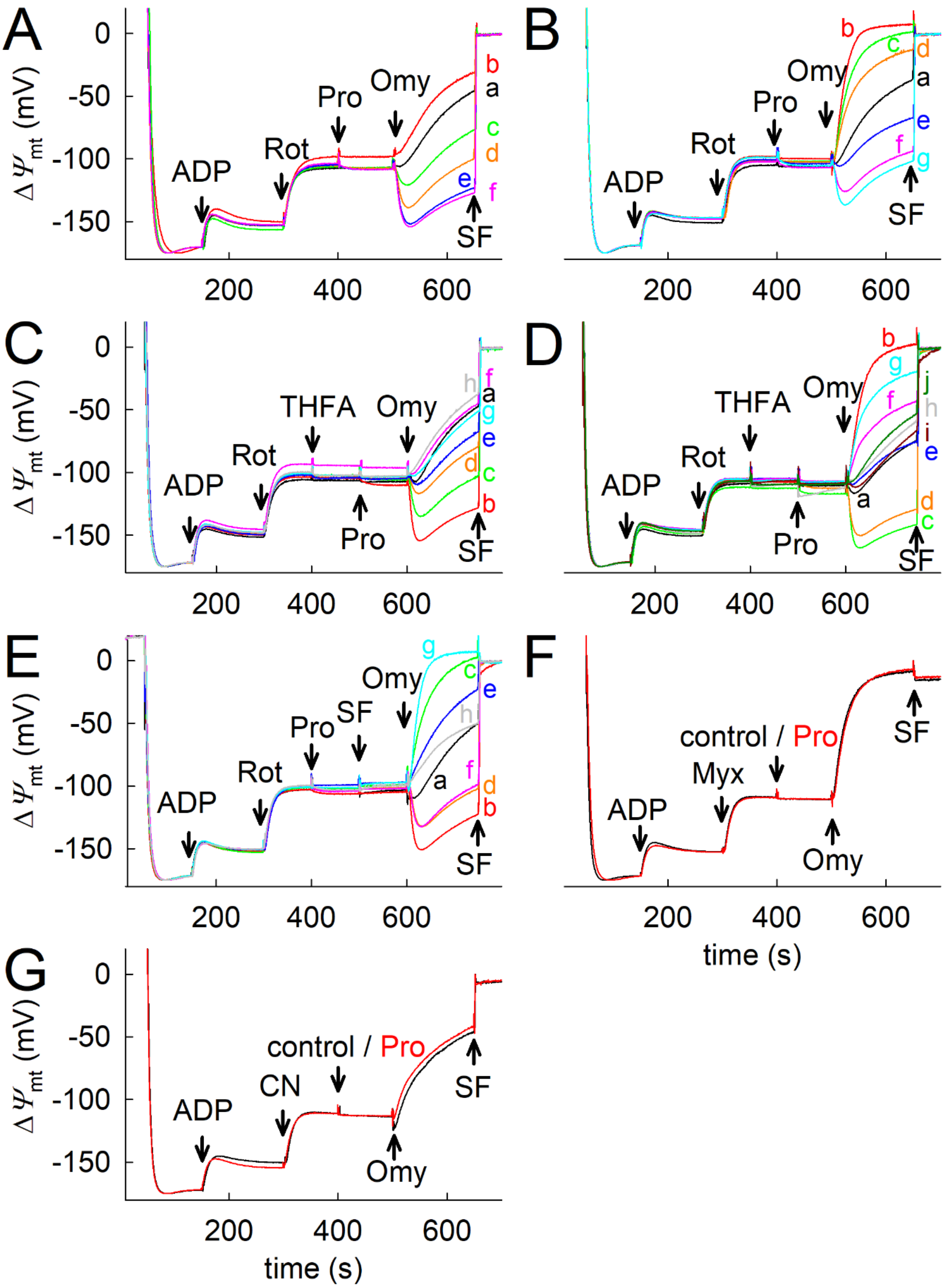

Supplementary Figure S12. The effect of proline on F_1F_0-ATPase directionality in isolated mouse kidney mitochondria as a function of targeted inhibition of electron transfer pathways using various substrate-inhibitor combinations. Traces are time courses of safranine O signal calibrated to $\Delta\Psi_{mt}$. **(A)** GM and increasing concentrations of Pro;

black (a): 0 mM; red (b): 0.25 mM; green (c): 0.5 mM; orange (d): 2 mM; blue (e): 5 mM; magenta (f): 10 mM. **(B)** GM (trace a) or GM & 5 mM itaconate (trace b to g), and increasing concentrations of Pro; black (a): 0 mM; red (b): 0 mM; green (c): 0.25 mM; orange (d): 0.5 mM; blue (e): 2 mM; magenta (f): 5 mM; cyan (g): 10 mM. **(C)** Black (a): GM; red (b): GM and 10 mM Pro; green (c): GM and 10 mM Pro and 2 mM THFA; orange (d): GM and 10 mM Pro and 5 mM THFA; blue (e): GM and 10 mM Pro and 10 mM THFA; magenta (f): GM and 2 mM THFA; cyan (g): GM and 5 mM THFA; grey (h): GM and 10 mM THFA. **(D)** Black (a): GM; red (b): GM & 5 mM itaconate; green (c): GM and 10 mM Pro; orange (d): GM & 5 mM itaconate and 10 mM Pro; blue (e): GM & 5 mM itaconate and 10 mM Pro and 2 mM THFA; magenta (f): GM & 5 mM itaconate and 10 mM Pro and 5 mM THFA; cyan (g): GM & 5 mM itaconate and 10 mM Pro and 10 mM THFA; grey (h): GM and 2 mM THFA; brown (i): GM and 5 mM THFA; dark green (j): GM and 10 mM THFA. **(E)** Black (a): GM; red (b): GM and 5 mM Pro; green (c): GM & 1 nM SF added @500 s; orange (d): GM & 1 nM SF added @500 s and 5 mM Pro; blue (e): GM & 2 nM SF added @500 s; magenta (f): GM & 2 nM SF added @500 s and 5 mM Pro; cyan (g): GM & 3 nM SF added @500 s; grey (h): GM & 3 nM SF added @500 s and 5 mM Pro. **(F)** myxothiazol (Myx); black trace: GM; red trace: GM and 10 mM Pro. **(G)** NaCN (CN); black trace: GM; red trace: GM and 10 mM proline.

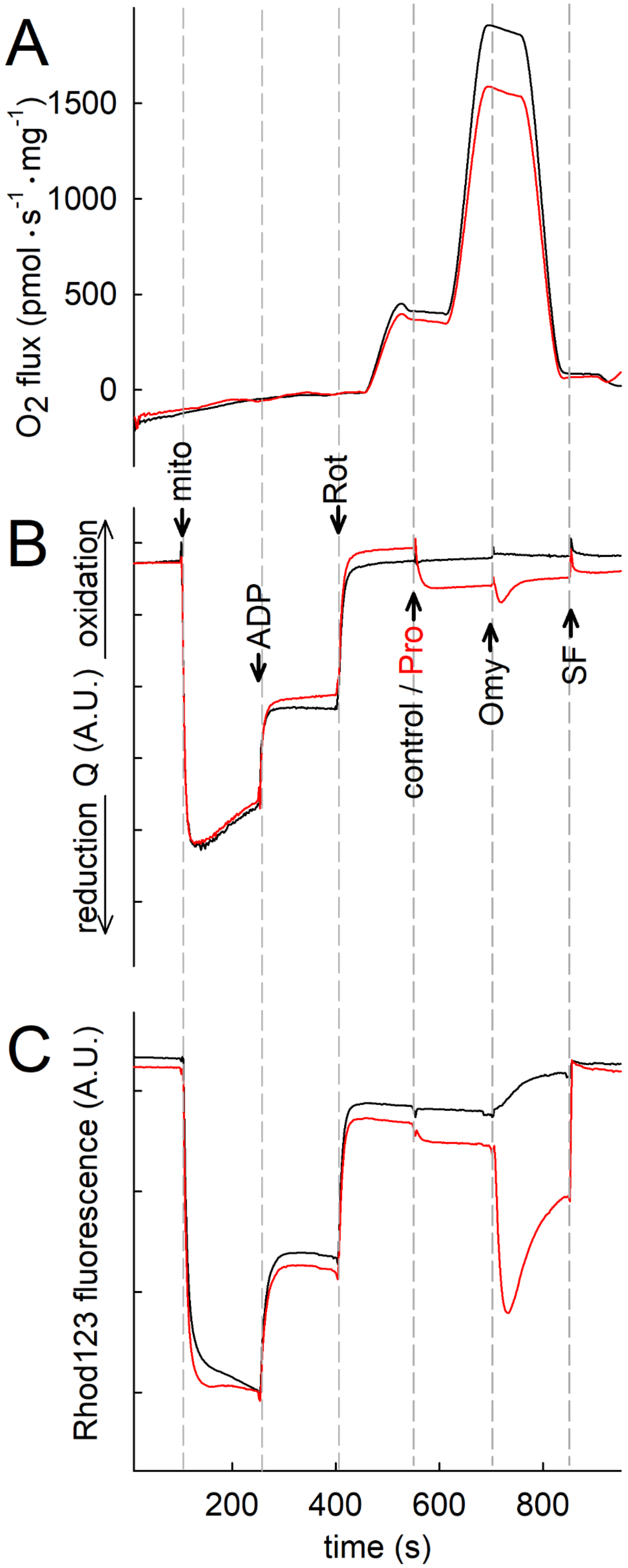

A
B
C
O2 flux (pmol·s-1·mg-1)
1500
1000
500
0
reduction Q (A.U.) oxidation
Rhod123 fluorescence (A.U.)
mito
ADP
Rot
control / Pro
Omy
SF
200
400
600
800
time (s)

Supplementary Figure S13. The effect of proline on oligomycin-induced changes in Q redox state. Time courses of oxygen consumption, Q redox state, and rhodamine 123 fluorescence indicative of $\Delta\Psi_{mt}$ (arbitrary units A.U.), respectively, recorded simultaneously from the same liver mitochondria using the NextGen-O2k and aligned on the dashed grey lines. 0.25 µM SF. Black trace: GM; red trace: GM and 5 mM Pro.
