# Supplementary figures and images for: Proline Oxidation Supports Mitochondrial ATP Production When Complex I Is Inhibited
Source: Int J Mol Sci. 2022 May 4;23(9):5111. doi: 10.3390/ijms23095111 (PMC9106064; doi:10.3390/ijms23095111)

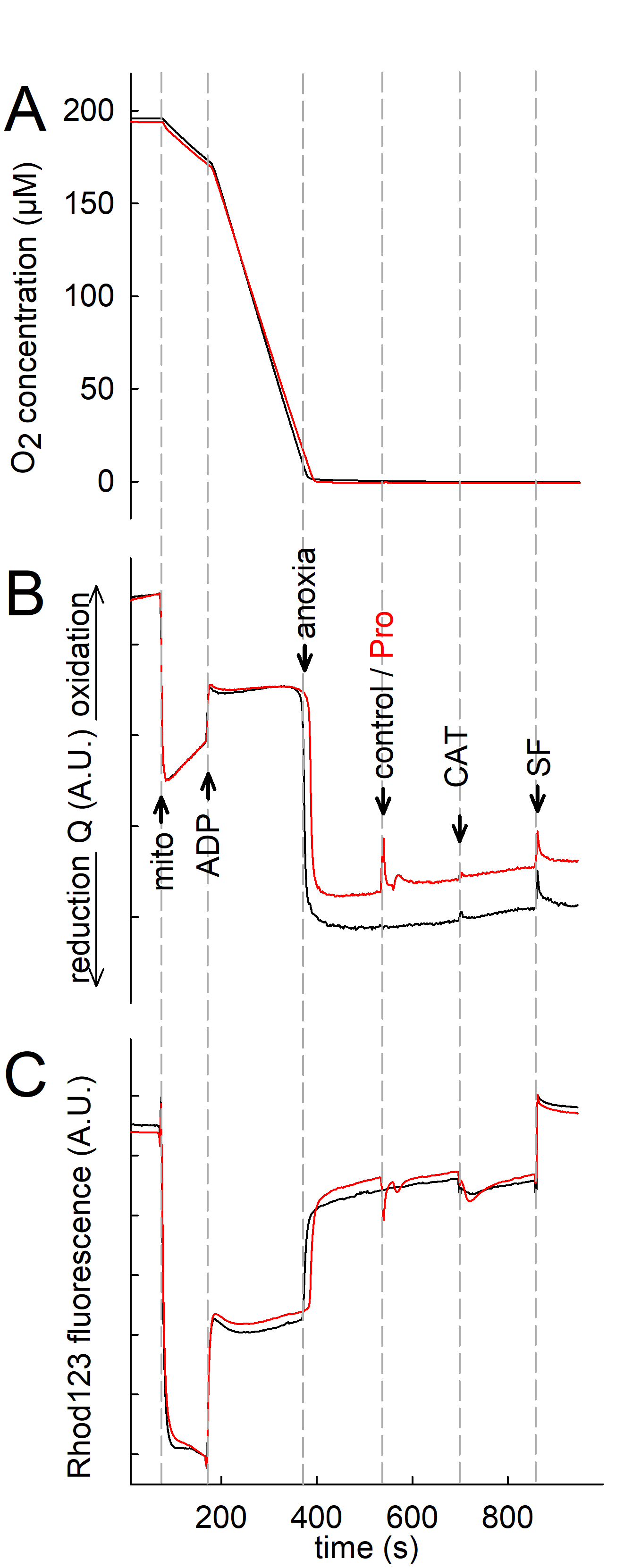

Supplement: Supplementary file 1 [file ijms-23-05111-s001.zip › supplementary figure 11.TIF]

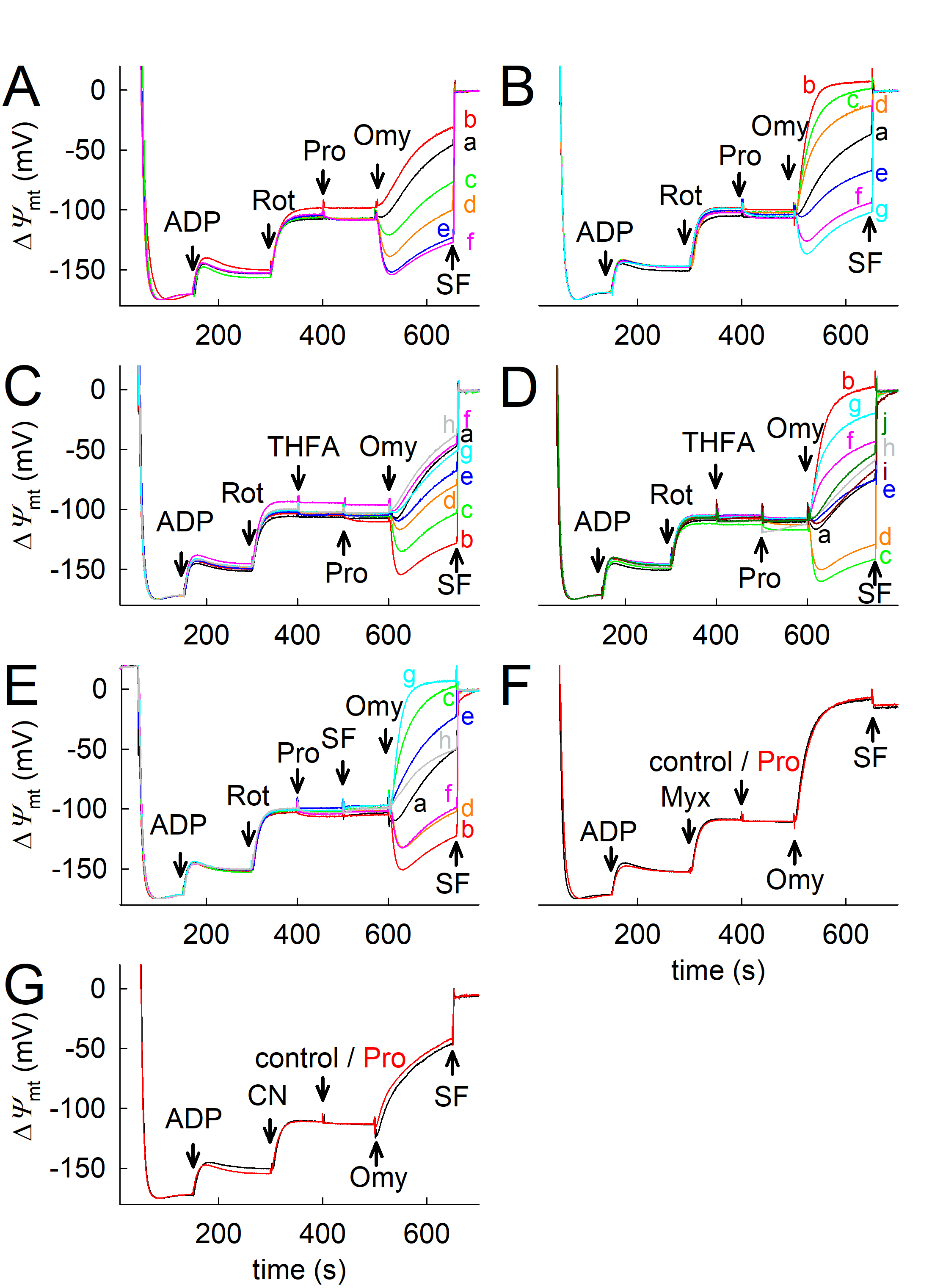

Supplement: Supplementary file 1 [file ijms-23-05111-s001.zip › supplementary figure 12.TIF]

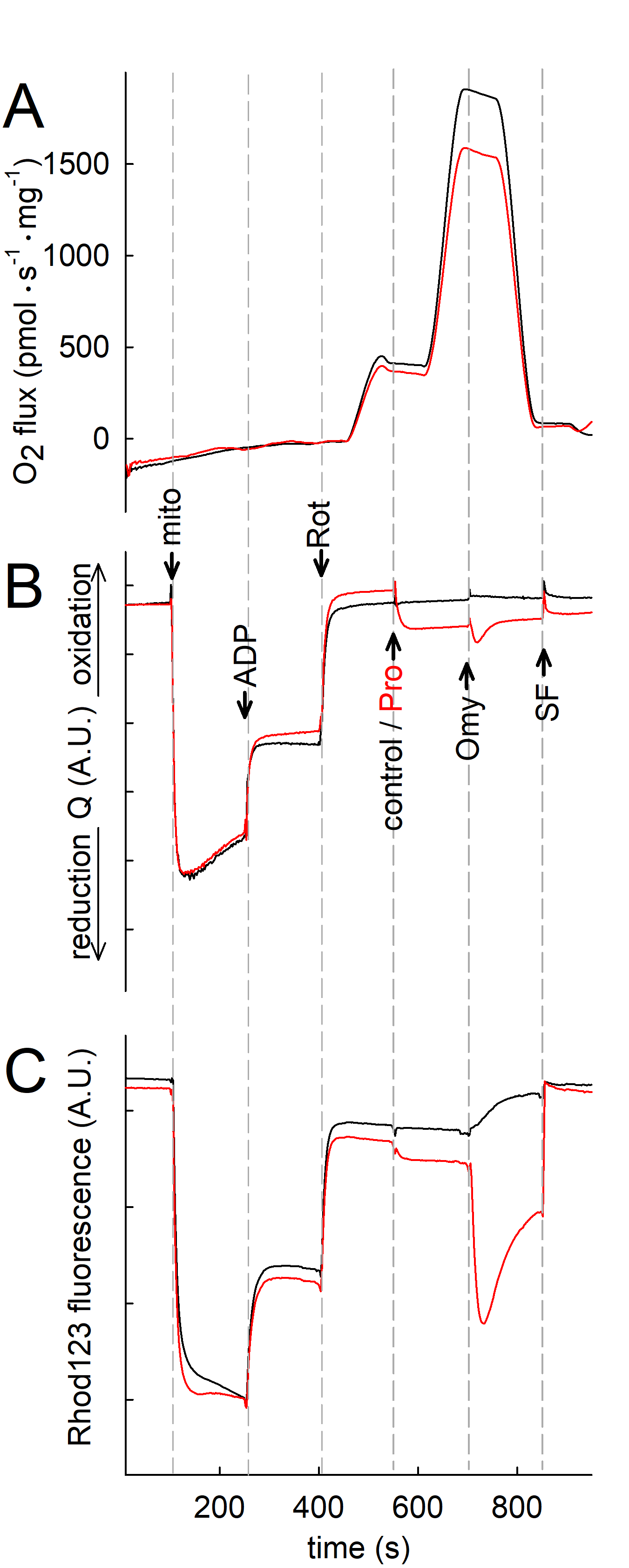

Supplement: Supplementary file 1 [file ijms-23-05111-s001.zip › supplementary figure 13.TIF]

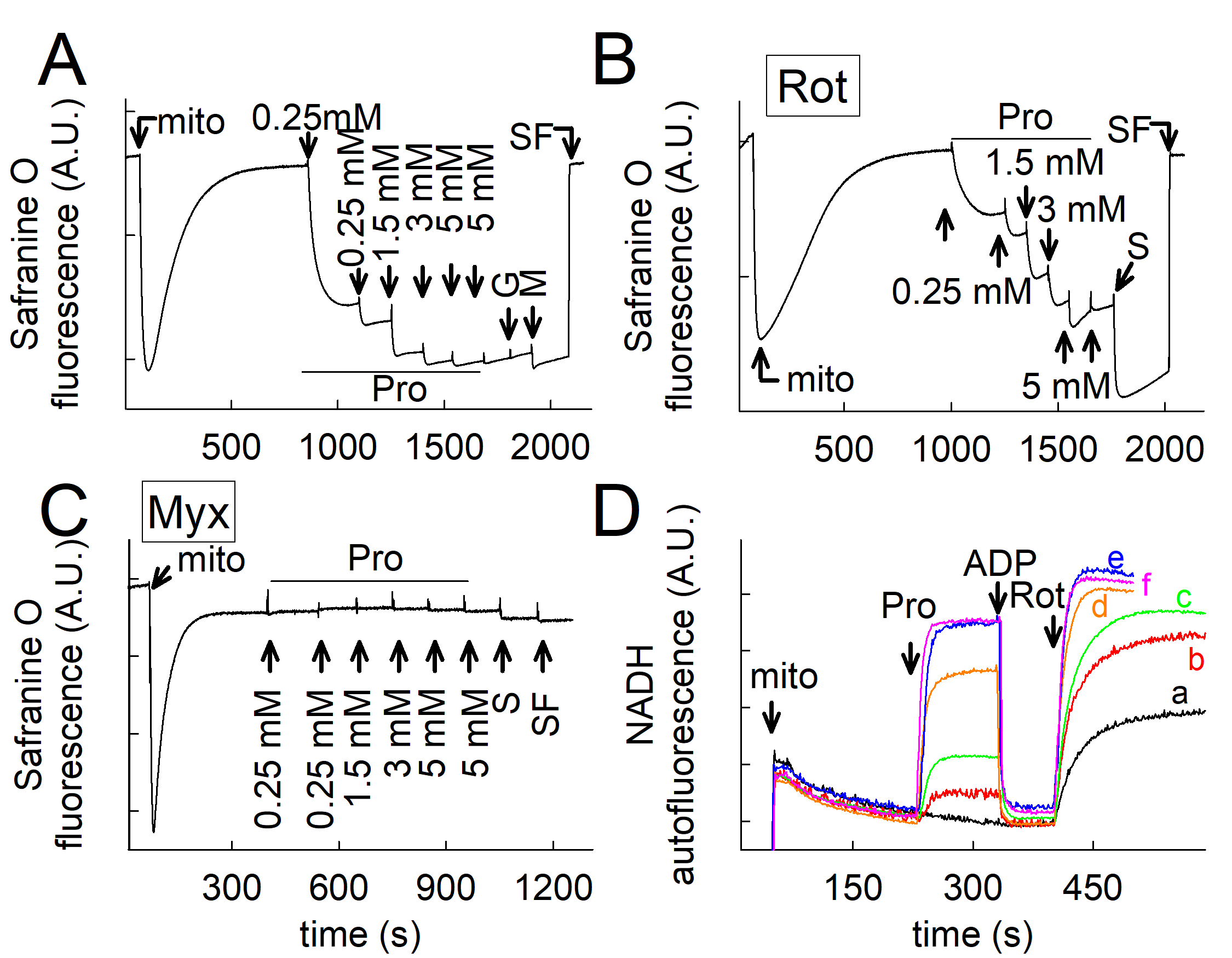

Supplement: Supplementary file 1 [file ijms-23-05111-s001.zip › supplementary figure S1.tiff]

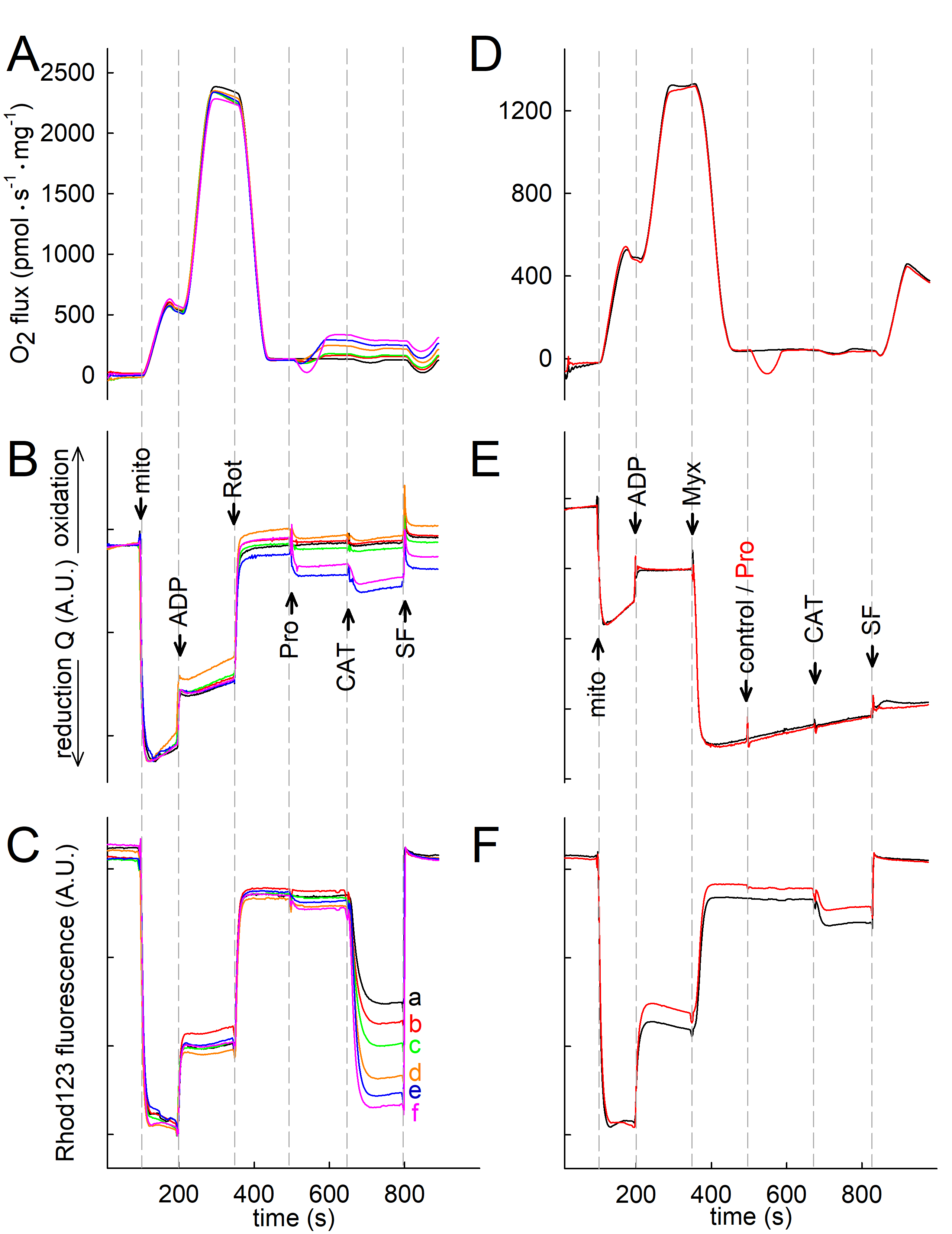

Supplement: Supplementary file 1 [file ijms-23-05111-s001.zip › supplementary figure S10.tiff]

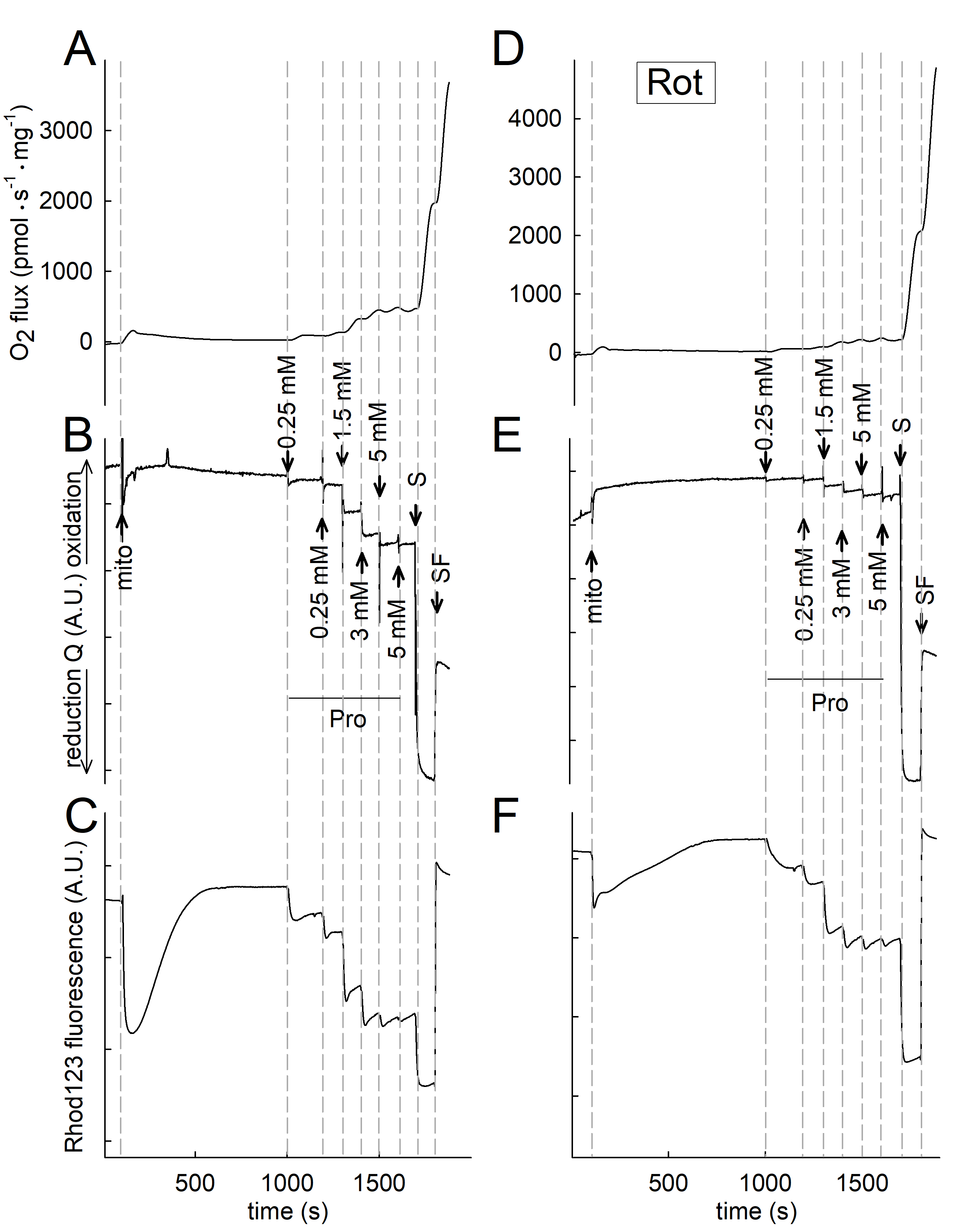

Supplement: Supplementary file 1 [file ijms-23-05111-s001.zip › supplementary figure S2.tiff]

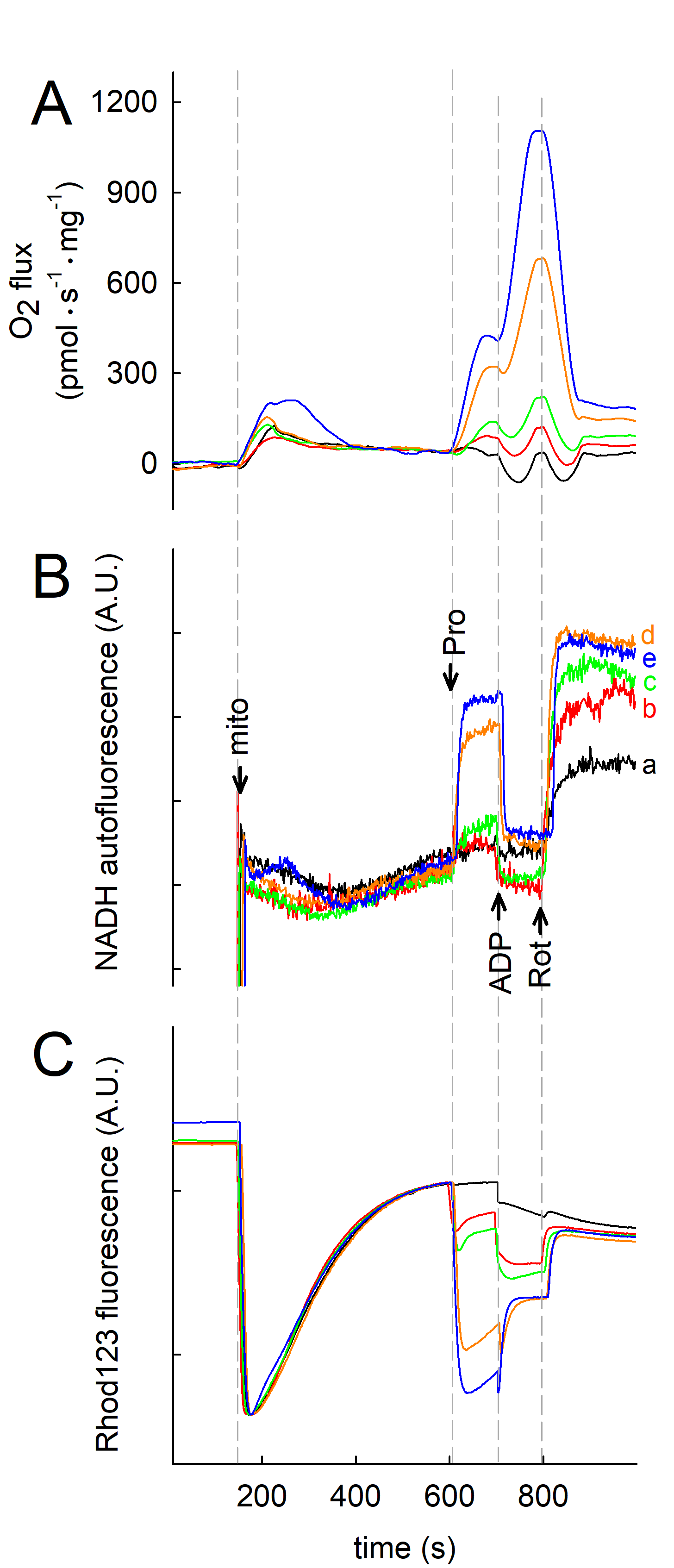

Supplement: Supplementary file 1 [file ijms-23-05111-s001.zip › supplementary figure S3.TIF]

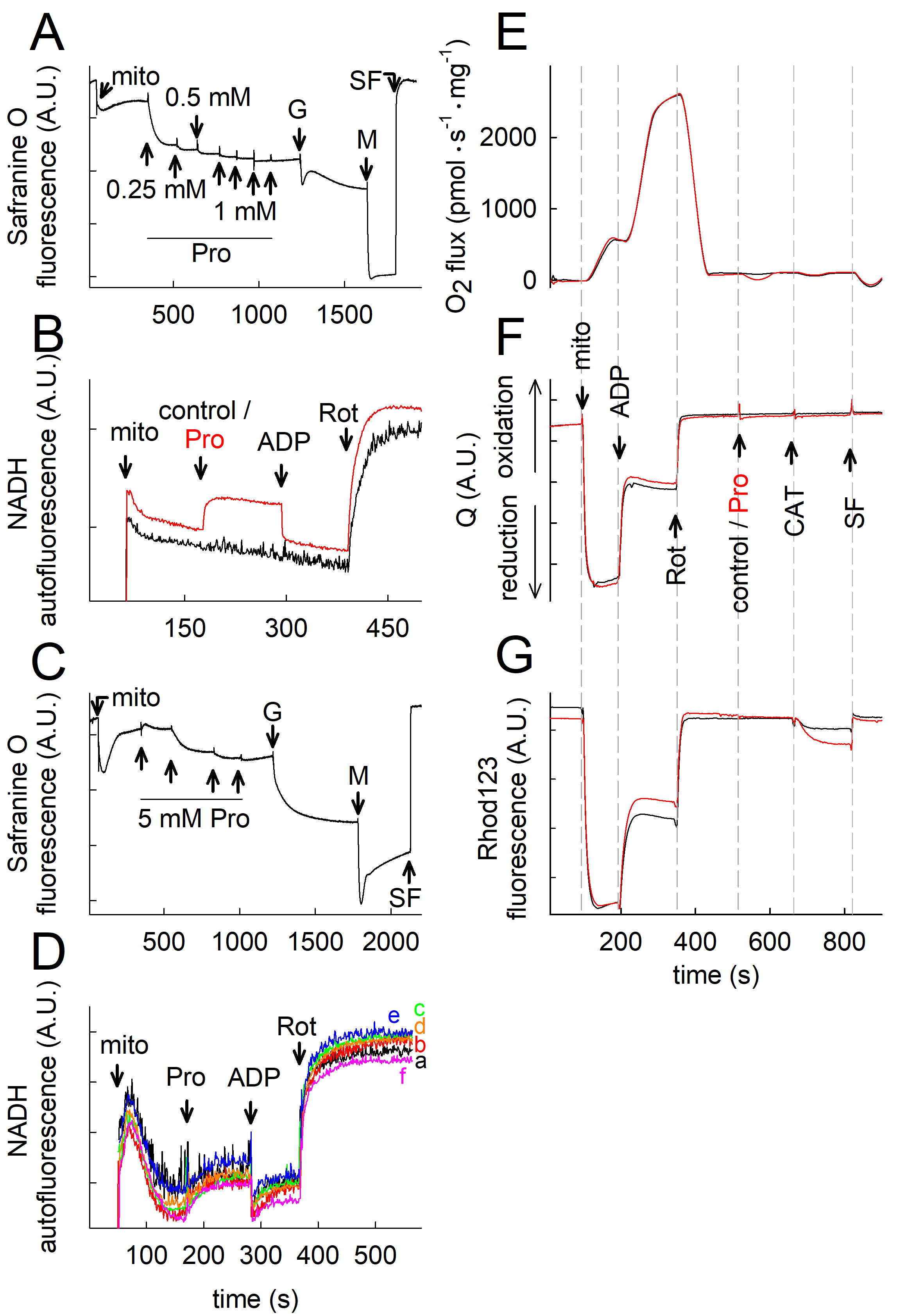

Supplement: Supplementary file 1 [file ijms-23-05111-s001.zip › supplementary figure S4.tiff]

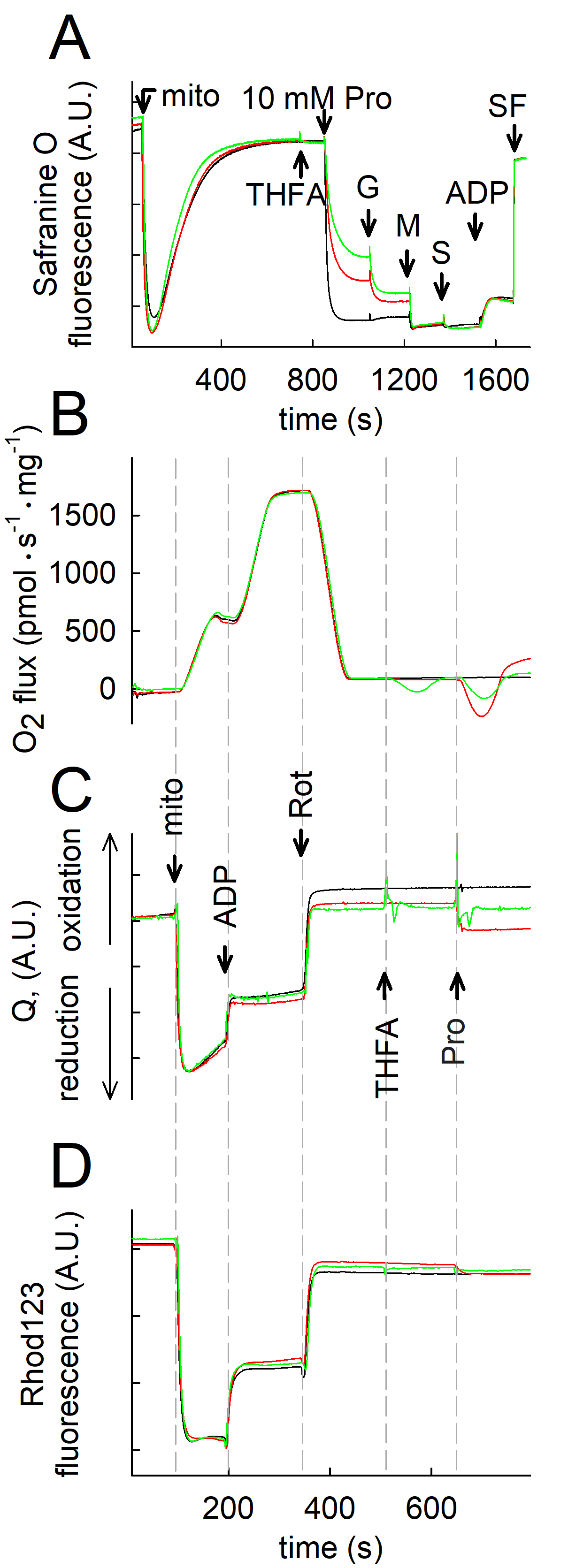

Supplement: Supplementary file 1 [file ijms-23-05111-s001.zip › supplementary figure S5.TIF]

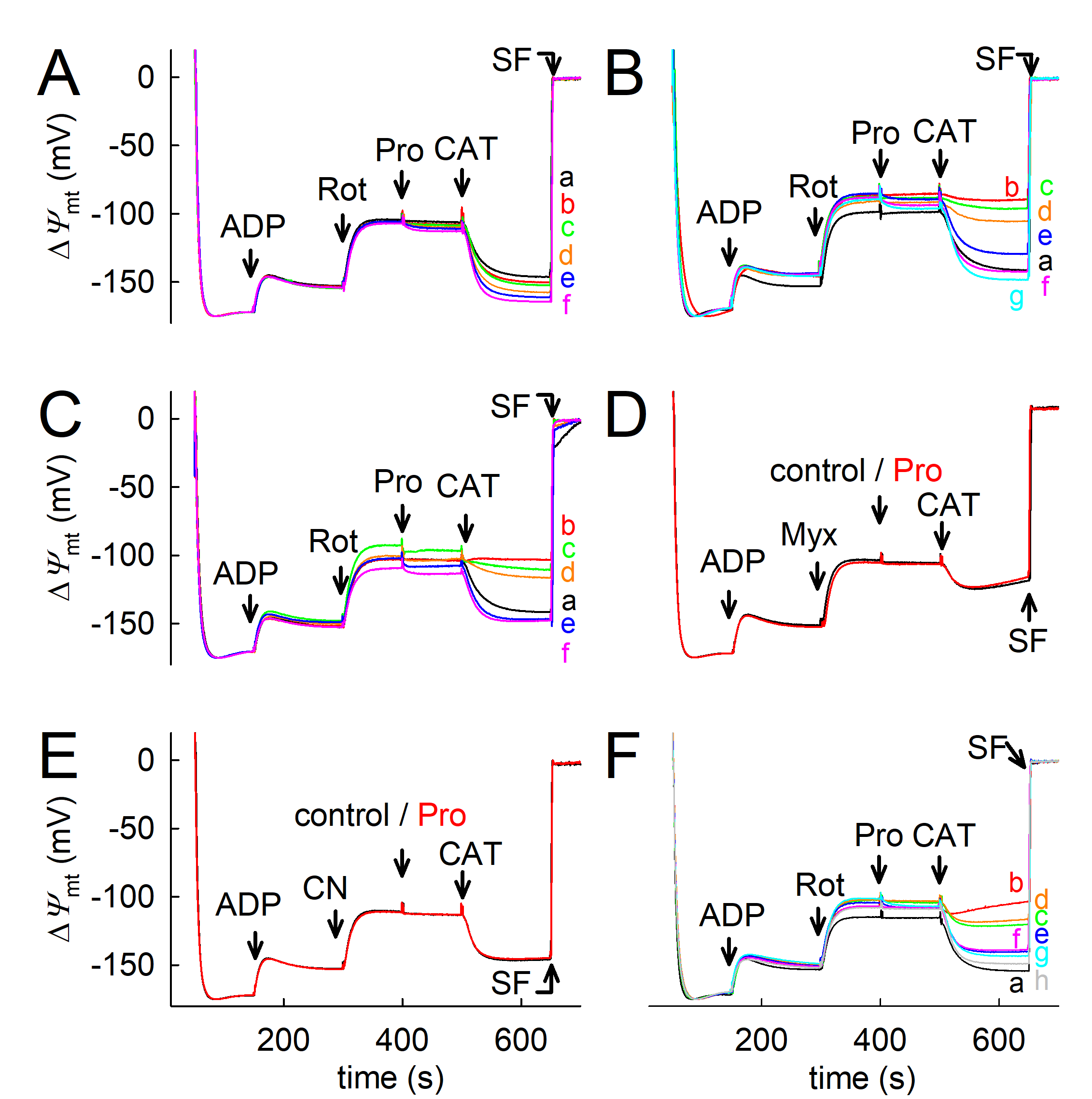

Supplement: Supplementary file 1 [file ijms-23-05111-s001.zip › supplementary figure S6.tiff]

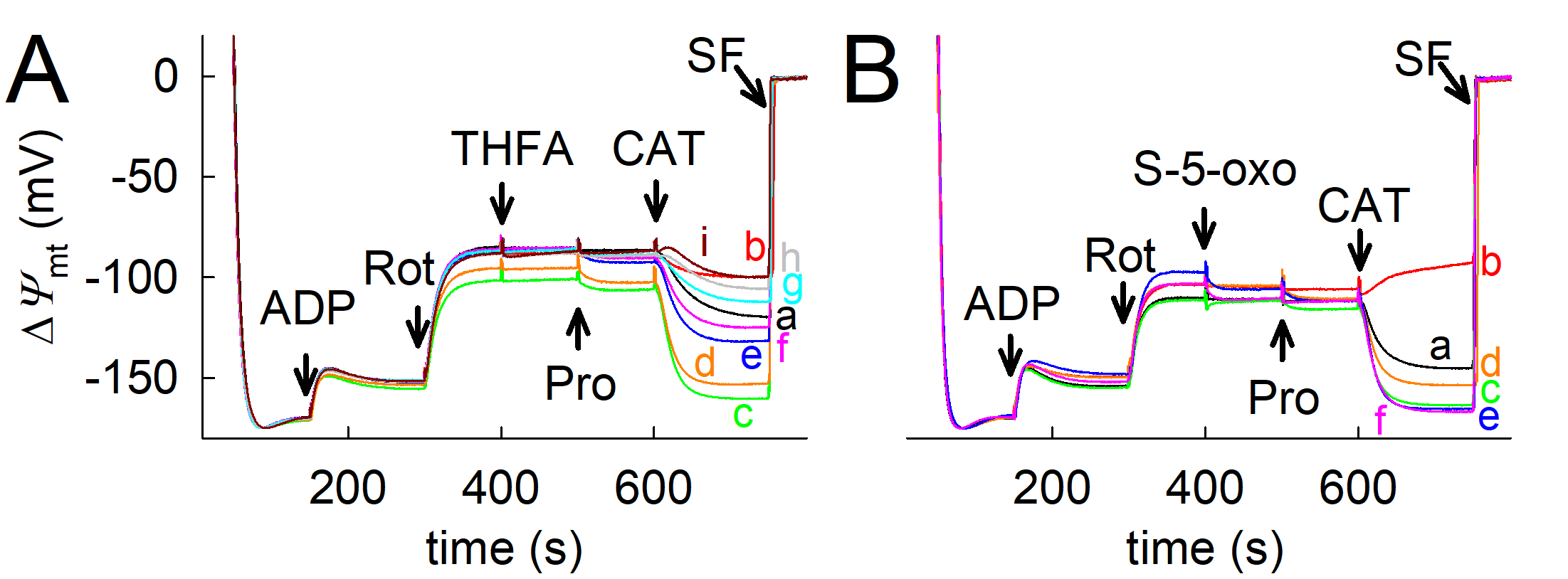

Supplement: Supplementary file 1 [file ijms-23-05111-s001.zip › supplementary figure S7.tiff]

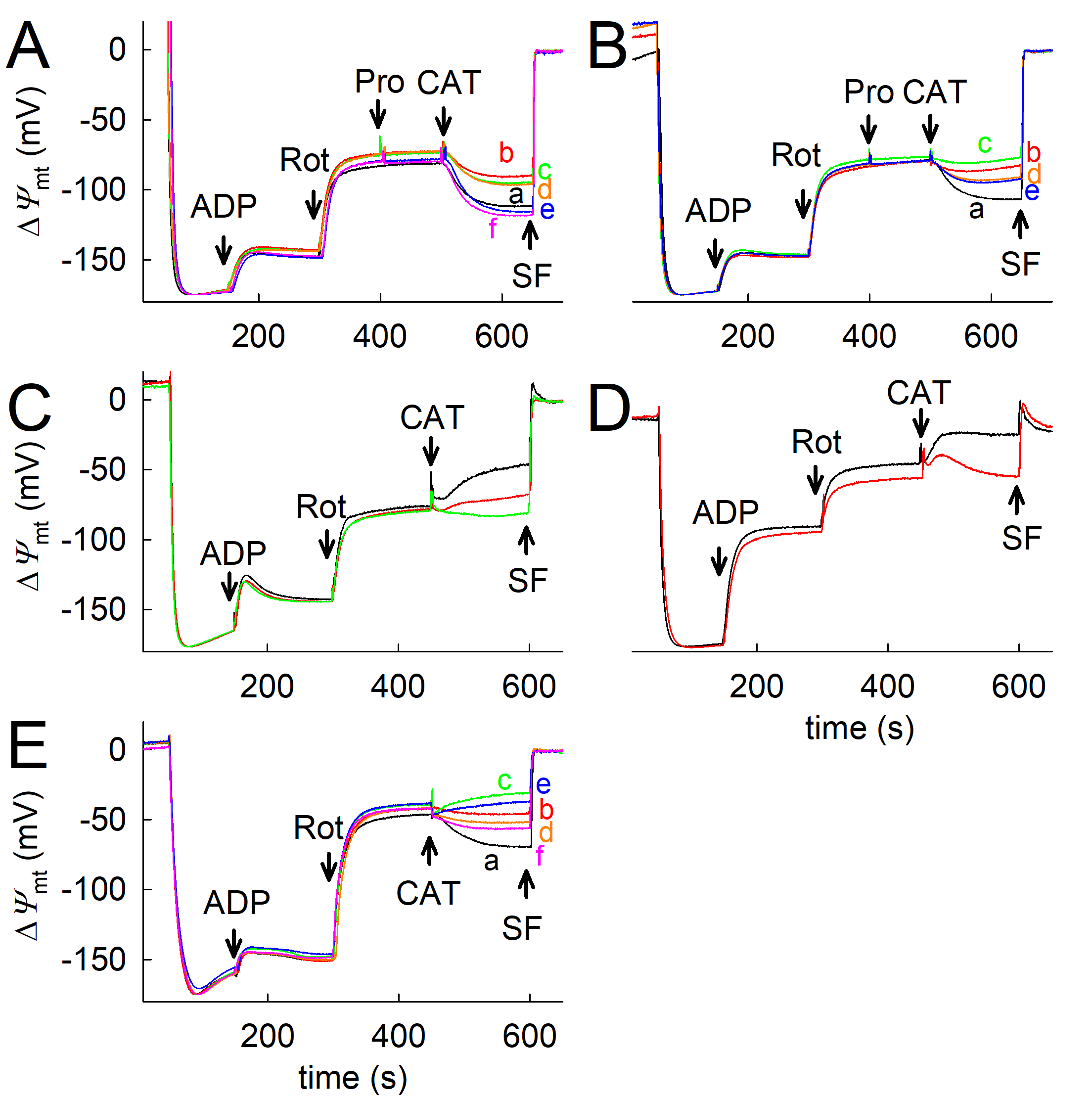

Supplement: Supplementary file 1 [file ijms-23-05111-s001.zip › supplementary figure S8.tiff]

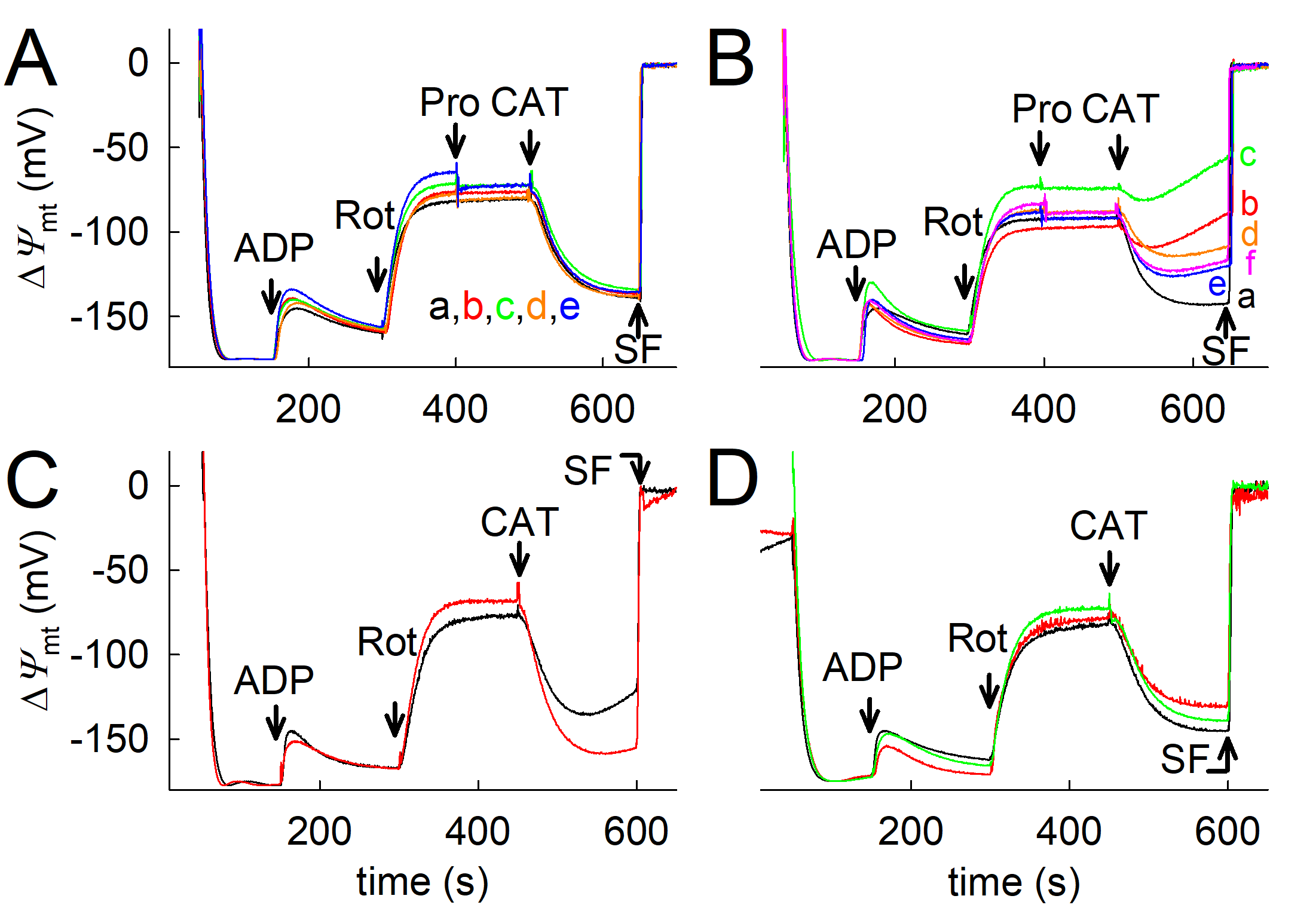

Supplement: Supplementary file 1 [file ijms-23-05111-s001.zip › supplementary figure S9.tiff]
